# Future Perspectives on the Application of Systems Biology and Generative Artificial Intelligence in the Design of Immunogenic Peptides for Vaccines

**DOI:** 10.3390/vaccines14020177

**Published:** 2026-02-13

**Authors:** José M. Pérez de la Lastra, Isidro Sobrino, Víctor M. Rodríguez Borges, José de la Fuente

**Affiliations:** 1 Biotechnology of Macromolecules, Instituto de Productos Naturales y Agrobiología, Consejo Superior de Investigaciones Científicas (IPNA-CSIC), Avda. Astrofísico Francisco Sánchez, 3, 38206 La Laguna, Spain; victor_mrb@yahoo.es; 2 Health and Biotechnology (SaBio), Instituto de Investigación en Recursos Cinegéticos (IREC), Consejo Superior de Investigaciones Científicas (CSIC), Universidad de Castilla-La Mancha (UCLM)-Junta de Comunidades de Castilla-La Mancha (JCCM), Ronda de Toledo 12, 13005 Ciudad Real, Spain; isidro.sobrino@uclm.es; 3 Department of Veterinary Pathobiology, Center for Veterinary Health Sciences, Oklahoma State University, Stillwater, OK 74078, USA

**Keywords:** generative artificial intelligence, peptide, vaccine, systems vaccinology, experimental validation, multi-epitope design, in silico–in vitro–in vivo integration

## Abstract

Peptide-based vaccines offer a modular and readily manufacturable platform for both prophylactic and therapeutic immunization. However, their broader translation has been constrained by the limited capacity to predict protective immunity directly from sequence-level features. Recent advances in systems vaccinology and high-throughput immune profiling have substantially expanded the experimental evidence, while generative artificial intelligence now enables de novo design of peptide immunogens and multi-epitope antigens under precisely controlled constraints. This review approaches how these complementary developments are transforming peptide vaccine research, moving beyond classical reverse vaccinology and conventional epitope prediction toward integrated, data-driven design frameworks. We discuss key generative model architectures and conditioning strategies aligned with vaccine objectives, including approaches that account for structural presentation, antigen processing and population-level human leukocyte antigen (HLA) diversity. Central to this perspective is the requirement for rigorous experimental validation and for strengthening the computational–experimental feedback loop through iterative in vitro and in vivo testing informed by systems-level immune readouts. We highlight representative applications spanning infectious diseases, cancer immunotherapy and vector-borne vaccinology, and we outline major technical and translational challenges that must be addressed to enable robust real-world deployment. Finally, we propose future directions for precision peptide vaccinology, emphasizing standardized functional benchmarks, the development of richer curated datasets linking sequence space to immune outcomes, and the early incorporation of formulation and delivery constraints into generative design pipelines.

## 1. Introduction

Vaccine development is entering a transformative phase driven by the convergence of immunology, high-throughput biology, and computational science. Traditional empirical vaccine discovery, which historically relied on labor-intensive cultivation and attenuation of pathogens, has been progressively replaced by rational, data-driven strategies that can markedly compress development timelines [1,2]. This shift was powerfully demonstrated during the COVID-19 pandemic, when the integration of computational modeling, structural biology, and rapid experimental validation enabled the design and deployment of highly effective vaccines within a single year [3,4,5].

Among modern vaccine platforms, peptide-based vaccines occupy a unique position. Peptide-based vaccines are particularly well suited for rational and computationally guided design because of their intrinsic modularity, chemical definability, and favorable safety profile. Unlike viral vectors or nucleic acid–based platforms, synthetic peptides are non-replicating, lack infectious potential, and can be manufactured with high purity and reproducibility using well-established chemical synthesis workflows. Compared with full-length protein antigens, peptides allow precise control over epitope composition, density, and immune targeting, enabling the selective inclusion or exclusion of specific T cell and B cell determinants. This level of granularity is especially advantageous for precision and population-tailored vaccination strategies, where human leukocyte antigen (HLA) diversity, antigen conservation, and immune polarization must be explicitly considered. At the same time, peptide vaccines naturally align with in silico and generative design paradigms, as their relatively small sequence space, well-defined structure–function relationships, and compatibility with multi-objective optimization make them highly amenable to de novo sequence generation and iterative experimental refinement [6,7,8]. Nevertheless, designing peptide immunogens capable of eliciting robust, durable, and protective immune responses remains a formidable challenge [9]. This perspective aligns with broader conceptual frameworks that view peptide vaccines as a flexible and future-oriented platform, capable of integrating advances in immunopeptidomics, systems immunology, and computational design to enable increasingly precise and personalized vaccination strategies [10].

The immunological effectiveness of a vaccine candidate emerges from complex, non-linear interactions involving antigen structure, host genetics, antigen processing and presentation pathways, and immune network dynamics [11]. Consequently, conventional immunoinformatic pipelines, centered primarily on epitope prediction, often struggle to anticipate real-world vaccine performance, particularly when immunogenicity is treated as a static molecular property rather than as an emergent biological process [1,12].

Classical reverse vaccinology has significantly advanced antigen discovery by enabling genome-wide screening of potential vaccine targets [13]. However, current pipelines typically reduce vaccine design to a sequence of filtering steps, including redundancy removal, homology exclusion, essentiality prediction, subcellular localization, and epitope mapping. While effective for narrowing candidate pools, these approaches often under-represent the systemic complexity of immune responses [12,14].

The distinction between antigenicity, immunogenicity, and protective efficacy further highlights this limitation. A peptide is antigenic if it can bind an immune receptor, either TCR or BCR, which implies the presence of epitopes specific to these receptors in its sequence and or structure [15]. If it can trigger an immune response on its own, which implies correct antigen presentation and or promotion of memory cell expansion, it is considered immunogenic [15]. A further layer is protective efficacy, namely the capacity to induce an immune response that is functionally protective, which depends on factors such as epitope context, antigen processing, immune cell cross-talk, and adjuvant effects [16,17]. Together, these considerations underscore the necessity of integrating computational prediction with experimental validation throughout the vaccine development process [18].

Recent advances in systems biology and artificial intelligence are redefining this landscape. The accumulation of high-resolution immunological data, encompassing immunopeptidomics, immune repertoire sequencing, and multi-omics profiling, now enables increasingly faithful modeling of host immune responses [4,19].

In parallel, generative AI architectures such as transformers, variational autoencoders, reinforcement-learning frameworks, and diffusion models are enabling the de novo design of peptide sequences under explicit immunological constraints, moving beyond simple epitope binding prediction [20,21,22]. Related advances in sequence design have further accelerated this transition by enabling high-throughput generation of sequences with predefined structural and functional constraints [23,24].

However, predictive power alone is insufficient for vaccine development. A central bottleneck remains the translation of computational outputs into biologically functional vaccines, motivating an in silico to in vitro to in vivo continuum in which computational design, experimental screening, and animal or clinical validation operate as an integrated feedback loop [4,25]. In this paradigm, experimental results continuously refine computational models, enabling progressively improved vaccine candidates grounded in measured immunological performance [2,26,27].

**Figure 1:**
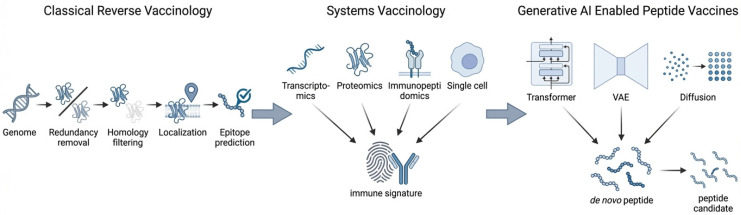
Conceptual transition from classical reverse vaccinology to integrated systems- and generative AI-guided peptide vaccine design. Classical reverse vaccinology relies on sequential genome-based filtering and epitope prediction. Systems vaccinology incorporates multi-omics immune profiling to identify immune signatures associated with protection. The integration of systems-level immune information with generative artificial intelligence enables de novo design of peptide vaccine candidates under explicit immunological and biophysical constraints.

In this review, we explore how the integration of generative AI, systems-level immunological data, and experimental validation is improving peptide-based vaccine design. We examine emerging computational architectures for controlled peptide generation, highlight the essential role of experimental verification in closing the computational biological gap, and illustrate how these approaches are being applied to infectious diseases, cancer immunotherapy, and vector-borne diseases, including anti-tick vaccine development. Figure 1 schematically summarizes the conceptual transition from classical reverse vaccinology toward integrated, systems-driven and generative design frameworks for peptide vaccine development.

## 2. From Classical Reverse Vaccinology to Systems-Guided Antigen Selection

The introduction of reverse vaccinology represented a paradigm shift in vaccine development by enabling genome-wide screening of potential antigens without the need to culture the pathogen [28]. This approach rapidly expanded the antigen discovery space and facilitated the development of several modern vaccines [29,30]. Traditional reverse vaccinology pipelines generally rely on a sequential series of computational filters, including removal of redundant sequences, exclusion of proteins homologous to host proteins, prediction of essential genes, estimation of subcellular localization, and identification of potential T and B cell epitopes [31,32,33].

While these pipelines remain highly valuable for reducing candidate complexity, their design reflects an implicit assumption that immunogenicity can be inferred from molecular features alone. In practice, however, vaccine efficacy is an emergent property of complex biological systems in which antigen structure, host genetic background, antigen processing, immune network dynamics, and environmental context interact in non-linear ways [1]. Consequently, purely sequence-driven selection strategies often show limited predictive power when translated into functional immune responses.

This limitation has become increasingly evident with the growth of immunological data. High-throughput experimental platforms, including mass spectrometry-based immunopeptidomics, single-cell transcriptomics, and immune repertoire sequencing, now provide quantitative and system-wide views of antigen processing and immune activation [34,35,36,37]. These technologies have revealed that epitope presentation, immunodominance and protective immunity cannot be reliably inferred from sequence motifs alone, but instead arise from coordinated multi-level interactions within the immune system [38,39]. For example, large scale immunopeptidomics studies in human and murine systems have demonstrated that only a limited fraction of computationally predicted high affinity peptides is naturally processed and presented on MHC molecules. Experimental analyses have shown that antigen presentation is strongly shaped by proteasomal processing, peptide transport and cellular context, often overriding simple binding affinity predictions, as illustrated in foundational studies by Gfeller and Bassani-Sternberg, and Abelin et al., which mapped thousands of naturally presented ligands and revealed substantial discrepancies between in silico prediction and in vivo presentation [40,41].

As a result, vaccine research is increasingly moving towards systems-guided antigen selection, where candidate antigens and peptides are evaluated not only by predicted binding or conservation, but also by their integration into immune response networks. Systems vaccinology approaches combine multi-omics measurements with computational modeling to identify immune signatures correlated with vaccine protection, durability, and safety [42,43,44,45]. This conceptual transition from reductionist filtering to network-informed design is schematically illustrated in Figure 1.

Beyond classical subtractive genomics and filtering pipelines, systems-guided antigen selection can also incorporate pathogen-level biology through network and constraint-based models. For example, protein–protein interaction networks enable prioritization of proteins with high centrality that are more likely to be essential, while flux balance analysis can identify conditionally essential metabolic genes under host-like environments. These approaches provide complementary signals for antigen prioritization that are not captured by sequence features alone and are especially relevant when protective mechanisms depend on perturbing critical pathogen functions [46,47,48].

Importantly, this systems perspective has profound implications for peptide-based vaccines. Rather than selecting isolated epitopes solely based on predicted affinity, modern design frameworks increasingly incorporate information on antigen processing, cytokine signaling pathways, immune cell cross-talk, and memory formation to guide peptide selection [49,50,51]. In this context, peptides are no longer treated as static molecular ligands but as dynamic components of immune regulatory networks whose behavior can only be fully understood through integrated computational and experimental analysis.

Thus, the transition from classical reverse vaccinology to systems-guided antigen selection establishes the conceptual foundation for next-generation peptide vaccine development. This shift provides the necessary framework for the subsequent introduction of generative artificial intelligence and closed computational–experimental design cycles, enabling the rational, data-driven design of effective peptide-based vaccines [52,53] (Table 1).

## 3. Antigenicity, Immunogenicity and Protective Efficacy: Moving Beyond Predictive Proxies

A central conceptual challenge in vaccine design lies in distinguishing between antigenicity, immunogenicity, and protective efficacy—three properties that are often conflated but fundamentally distinct (Table 2). First, a peptide is antigenic if it is capable of binding to an immune receptor, either T-cell receptor (TCR) or B-cell receptor (BCR), which necessarily implies the presence, in its sequence and/or structure, of epitopes specific to these receptors [15]. If, in turn, it is capable of triggering an immune response on its own—implying correct antigen presentation and/or the activation and expansion of memory cells—it is considered immunogenic [15]. An additional and critical layer is that of a protective antigen, defined as one that induces an effective and durable immune response capable of preventing infection or disease progression [62].

Although computational vaccinology has made remarkable progress in predicting antigenicity and, to some extent, immunogenicity, protective efficacy remains far more difficult to infer. This is because protection emerges from complex, non-linear interactions between antigen properties, antigen processing and presentation pathways, immune cell networks, and host genetic background [11,63]. As a consequence, many peptide candidates predicted to be strong binders or immunogenic in silico ultimately fail to confer meaningful protection in vivo.

Most existing immunoinformatic tools focus primarily on receptor binding, particularly MHC-peptide affinity, which is a necessary but not sufficient condition for effective vaccination [64]. Protective immunity further depends on additional parameters, including epitope density, antigen persistence, cytokine milieu, immune cell recruitment, and the formation of long-lived memory populations [65,66,67,68]. These parameters are not intrinsic properties of the peptide sequence itself but emerge from the dynamic behavior of the immune system as a whole.

This discrepancy between predicted and observed vaccine performance highlights a fundamental limitation of purely computational pipelines [69]: immunogenicity cannot be treated as a static molecular attribute but must instead be understood as a systems-level phenomenon. Consequently, predictive models must be complemented by experimental validation at multiple biological scales. In vitro assays—such as MHC binding, antigen processing, T-cell activation, and cytokine profiling—provide essential functional readouts, while in vivo models remain indispensable for assessing protective efficacy, safety, and durability of immune responses [70,71,72].

Several experimental studies illustrate this disconnect between predictive proxies and protective immunity. For instance, large-scale analyses of viral and tumor epitopes have shown that many peptides with high predicted MHC binding affinity fail to elicit functional T-cell responses in vivo, whereas some naturally dominant protective epitopes display only moderate predicted affinity. In influenza models, studies by Assarsson et al. and Yewdell et al. demonstrated that immunodominant and protective epitopes cannot be reliably inferred from binding strength alone, but depend strongly on antigen processing and cellular context [73,74]. Similarly, in cancer immunotherapy, multiple neoantigen vaccination trials have reported that only a subset of computationally prioritized peptides induce durable tumor-specific T-cell responses and clinical benefit, despite broad predicted immunogenicity in silico [75,76]. These observations underscore that protective efficacy emerges from system-level immune dynamics rather than from peptide properties in isolation [77].

In the context of peptide-based vaccines, this conceptual shift has major implications. Rather than selecting peptides solely on predicted binding scores, modern vaccine development increasingly emphasizes the integration of computational prediction with experimental immunology in iterative feedback loops. These closed-loop frameworks allow candidate peptides to be continuously refined based on measured biological performance, thereby bridging the long-standing gap between in silico design and protective immunity [78].

## 4. Generative Artificial Intelligence for Peptide and Multi-Epitope Vaccine Design

**Figure 2:**
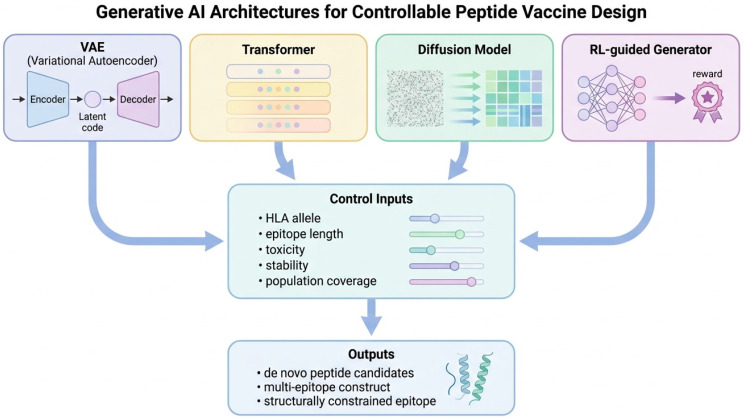
Generative artificial intelligence architectures for controllable peptide vaccine design. Schematic overview of representative generative model classes applied to peptide vaccine development, including variational autoencoders (VAEs), transformer-based models, diffusion models, and reinforcement learning-guided generators. While these architectures differ in how peptide sequence space is represented and explored, they can be conditioned on shared control inputs such as HLA allele specificity, epitope length, toxicity, stability, and population coverage. Model outputs include de novo peptide candidates, multi-epitope constructs, and structurally constrained epitopes, enabling flexible and multi-objective vaccine design.

The application of generative artificial intelligence (AI) represents a fundamental shift in vaccine design, moving the field from predictive screening toward the de novo creation of optimized immunogens. Unlike traditional immunoinformatic pipelines that evaluate existing sequences, generative models learn the underlying distribution of functional biological sequences and generate novel peptide candidates under explicitly controlled conditions. This capability is particularly transformative for peptide-based vaccines, where the combinatorial explosion of possible sequences makes exhaustive experimental exploration impossible. Figure 2 summarizes the main generative AI architectures and conditioning strategies currently applied to peptide and multi-epitope vaccine design.

### 4.1. Generative Architectures for Controlled Peptide Design

Several classes of generative models have demonstrated strong potential for peptide vaccine development. Variational autoencoders (VAEs) map peptide sequences into continuous latent spaces that enable smooth interpolation between functional regions and allow immunological properties to be tuned by navigating these latent dimensions. Autoregressive transformer-based language models learn long-range dependencies between amino acids and can generate peptides conditioned on length, composition, structural context, and immunological constraints [79]. Reinforcement learning frameworks further refine these models by optimizing multi-objective reward functions that incorporate predicted MHC binding, immunogenicity, solubility, stability, and toxicity. Although these model classes may appear to share similar inputs and outputs at a schematic level, they differ fundamentally in how sequence space is represented, constrained, and explored, with important consequences for controllability, diversity, and the integration of structural and immunological priors.

More recently, diffusion-based models have emerged as powerful tools for protein and peptide design. These architectures generate sequences by iteratively transforming noise into structured biological molecules while enforcing geometric and biophysical constraints [80].

When coupled with structure-aware sequence optimization methods such as ProteinMPNN [81], diffusion models enable the generation of peptides that are not only immunologically relevant but also structurally stable and manufacturable—an essential requirement for translational vaccine development.

### 4.2. Designing Multi-Epitope Vaccines with Generative AI

Generative AI is particularly well suited for the design of multi-epitope peptide vaccines, which combine multiple immunogenic regions to enhance population coverage and immune robustness. From a design perspective, it is important to distinguish between two related but conceptually distinct use cases for generative models in multi-epitope vaccine development. In one scenario, generative frameworks are used to optimize sets of individual peptides intended to be administered as cocktails, without explicit sequence linkage. In this case, models focus primarily on epitope selection, population coverage, and functional complementarity. In a second scenario, generative models are tasked with designing fully linked multi-epitope constructs, often referred to as “beads-on-a-string” vaccines, in which epitope ordering, linker composition, and overall sequence context can strongly influence antigen processing, presentation efficiency, and immunodominance [38,39,40,41,73,74]. These two settings impose different modeling requirements and rely on different types of training data. While datasets supporting peptide-level optimization are relatively abundant, experimentally validated data linking specific epitope orderings or linker designs to immunogenic outcomes remain scarce. As a result, current generative approaches for linked constructs often rely on transfer learning from protein design, immunopeptidomics, and antigen processing studies, and should be viewed as an emerging area with significant data-driven limitations. Rather than assembling epitope constructs heuristically, generative frameworks can simultaneously optimize epitope selection, ordering, linker composition, and structural presentation. These models can explicitly account for antigen processing, epitope competition, and immune dominance effects, thereby reducing the risk of construct interference and suboptimal immune responses [73,74].

A recurrent practical limitation of conventional multi-epitope constructs is that linear “string-of-beads” designs can remain structurally flexible and unstable, which may compromise epitope presentation and promote aggregation or unintended immunodominance. This motivates structure-aware design strategies in which epitopes are embedded within more structurally constrained scaffolds, enabling better control of epitope spacing, orientation and overall manufacturability [82,83].

Importantly, generative models enable the rational design of peptide scaffolds that stabilize epitopes in conformations favorable for B-cell and T-cell recognition. Structure-guided generation using diffusion architectures allows epitopes to be presented on custom-designed protein frameworks, mimicking native antigen geometries and enhancing neutralizing antibody induction [80]. This approach opens new possibilities for vaccine formulations targeting conformational epitopes that are inaccessible to traditional linear peptide vaccines.

### 4.3. Integrating Immunological Constraints and Vaccine Performance Objectives

A key advantage of generative AI in vaccinology is its ability to incorporate explicit immunological constraints into the design process. Models can be conditioned on HLA allele distributions, population coverage, antigen conservation, predicted cytokine profiles, and safety parameters. By embedding these constraints into the generative objective function, AI systems generate peptides that are pre-optimized for real-world vaccine deployment.

This capacity for controlled generation supports emerging paradigms of precision vaccinology, in which vaccine formulations are tailored to specific populations, host genotypes, or disease contexts [84]. For example, in oncology, generative and AI-assisted computational frameworks are increasingly used to support the design of patient-specific neoantigen vaccines, while in infectious disease research similar approaches facilitate the rapid adaptation and redesign of vaccine candidates in response to pathogen evolution and antigenic variation [75,76,85,86].

### 4.4. Toward Structurally Programmed Peptide Vaccines

The convergence of generative AI with structural vaccinology is driving the development of structurally programmed peptide vaccines. By integrating cryo-electron microscopy-derived structural information with protein structure prediction and generative design frameworks, researchers can engineer immunogens in which epitope geometry and spatial presentation are explicitly constrained and experimentally grounded, enabling more realistic and controllable immunogen design [80,81]. These advances enable the construction of self-assembling nanoparticle vaccines and epitope-focused scaffolds that enhance immune recognition and promote durable protective responses.

Together, these developments illustrate how generative AI transforms peptide-based vaccine design from a screening problem into an engineering discipline. Rather than searching for suitable peptides within existing proteomes, vaccine developers can now design immunogens directly, guided by immunological objectives, structural constraints, and experimental feedback (Table 3). It should be noted that the capabilities summarized below are not exclusive to any single model class, but rather reflect design patterns and strengths that can be realized depending on model architecture, parametrization, training data, and objective formulation.

This transition fundamentally redefines peptide vaccine development as a controllable engineering problem rather than a purely empirical discovery process.

## 5. Experimental and Computational Integration: Closing the In Silico–In Vitro–In Vivo Loop

**Figure 3:**
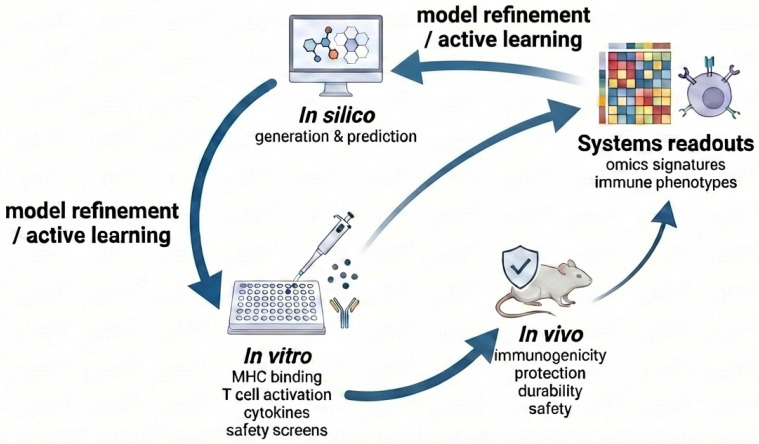
Integrated in silico–in vitro–in vivo design loop for peptide vaccine development. Generative and predictive models propose candidate peptides in silico, which are evaluated through in vitro functional assays and in vivo immunogenicity, protection, durability, and safety studies. Systems-level immunological readouts, including omics signatures and immune phenotypes, are generated during experimental evaluation and fed back into the in silico stage to support model refinement and active learning across iterative design–test–learn cycles.

Despite the growing sophistication of computational models for antigen and peptide design, vaccine development ultimately depends on experimental validation (Figure 3).

**Figure 4:**
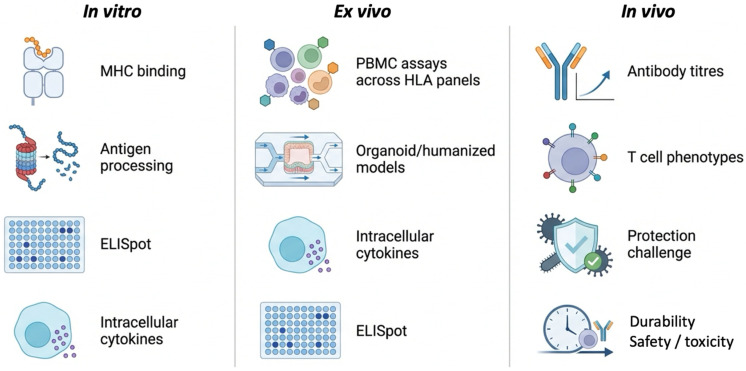
Experimental validation endpoints for AI-designed peptide vaccines. Overview of key functional assays and biological readouts used to validate computationally designed peptide vaccine candidates across the in vitro, ex vivo, and in vivo stages.

Predictive accuracy alone cannot guarantee protective immunity. The functional relevance of a vaccine candidate emerges only when computational design is systematically integrated with molecular, cellular, and organismal experimentation. This realization has given rise to the in silico–in vitro–in vivo continuum, an iterative framework that lies at the core of modern systems vaccinology [87,88]. Accordingly, in vivo evaluation encompasses not only immunogenicity and protective efficacy, but also durability of immune responses and safety or toxicity assessment, as summarized in Figure 4.

### 5.1. In Silico Design and Prioritization

At the computational stage, generative and predictive models propose large libraries of candidate peptides optimized for predefined immunological and biophysical criteria. These criteria typically include predicted MHC class I and II binding, antigen processing efficiency, population coverage, structural stability, solubility, toxicity, and manufacturability. Increasingly, multi-omics datasets—such as immunopeptidomics, transcriptomics, and immune repertoire sequencing—are incorporated to refine candidate selection based on systems-level immune behavior [89]. This integration ensures that candidate prioritization reflects observed immune behavior rather than purely theoretical predictions. By integrating these heterogeneous datasets, computational pipelines generate ranked candidate lists that balance immunogenic potential with translational feasibility. However, these predictions remain hypotheses until experimentally verified.

Importantly, this stage also marks a conceptual departure from classical in silico vaccine pipelines, which primarily rely on ranking and filtering epitopes derived from existing proteomes. In contrast, generative models shift the design paradigm by directly proposing novel peptide sequences and multi-epitope constructs that satisfy predefined immunological and biophysical constraints. Rather than asking which naturally occurring peptides score highest according to a given metric, generative frameworks ask which sequences should exist to optimally achieve a desired immune outcome. This transition from candidate selection to candidate creation is central to the emerging role of generative artificial intelligence in peptide vaccine development and directly underpins the perspective advanced in this review [20,21,22].

### 5.2. In Vitro Functional Validation

In vitro experimentation provides the first critical biological filter. Candidate peptides are evaluated using assays that directly measure immune-relevant functions, including MHC-peptide binding assays, antigen processing and presentation studies, T-cell activation assays, cytokine profiling, and toxicity screening. These assays establish whether predicted properties translate into measurable immunological activity under controlled conditions.

Such experimental data serve two essential purposes: they identify promising vaccine candidates for further development and they provide ground-truth labels for retraining and refining computational models. This feedback transforms the design process into a learning system in which predictions improve with each experimental cycle.

### 5.3. In Vivo Immunogenicity and Protective Efficacy

While in vitro assays capture essential molecular and cellular responses, in vivo models remain indispensable for assessing protective immunity, safety, and durability. Animal studies evaluate the capacity of peptide vaccines to elicit neutralizing antibodies, cytotoxic T-cell responses, immunological memory, and protection against challenge. These experiments also reveal potential adverse effects and immunopathological risks that cannot be detected in vitro.

Importantly, in vivo outcomes frequently expose discrepancies between predicted immunogenicity and actual protective efficacy, underscoring the necessity of maintaining the experimental arm of vaccine development at the center of the design loop.

### 5.4. Systems-Level Feedback and Model Refinement

The defining feature of the in silico–in vitro–in vivo framework is the continuous feedback of experimental data into computational models. High-dimensional immunological readouts—including cytokine profiles, immune cell phenotypes, and transcriptomic signatures—are integrated with peptide sequence and structural data to identify correlates of protection and failure [60,90].

This feedback enables generative AI systems to progressively learn which molecular and structural features truly drive protective immunity. Over successive design–test–learn cycles, vaccine candidates become increasingly optimized, while the design space is explored more efficiently.

### 5.5. Toward Autonomous Vaccine Design Pipelines

As computational models and experimental platforms become more tightly coupled, vaccine development is beginning to incorporate elements of semi-autonomous discovery, particularly at the in vitro screening stage. In these emerging workflows, generative models propose candidate peptides, automated or high-throughput experimental assays evaluate selected functional properties, and the resulting data are fed back to update model parameters through active learning strategies. Such partially closed-loop systems have already been explored in adjacent areas of drug discovery and protein engineering, where iterative design–test–learn cycles can substantially accelerate candidate optimization.

However, it is important to emphasize that full autonomy across the entire in silico–in vitro–in vivo continuum remains unrealistic at present. In vivo experimentation introduces biological complexity, ethical constraints, and time scales that cannot be readily automated, and therefore represents a fundamental bottleneck in closed-loop vaccine development. In practice, in vivo studies are likely to remain sparse, hypothesis-driven, and strategically placed at later stages of candidate refinement rather than incorporated into rapid iterative loops.

Within these constraints, the most realistic near-term impact of semi-autonomous pipelines lies in improving early-stage candidate quality and reducing experimental attrition before in vivo testing. For peptide-based vaccines in particular, the ability to rapidly generate, screen, and refine immunogens in vitro creates valuable opportunities to respond to emerging pathogens, evolving variants, and patient-specific targets, while maintaining rigorous experimental oversight [26,80]. Within these practical constraints, Table 4 summarizes the principal stages and experimental decision points that structure semi-integrated design–test–learn workflows in peptide vaccine development.

## 6. Case Studies and Applications: From Infectious Disease to Cancer and Vector-Borne Vaccinology

It is important to note that, despite rapid methodological advances, relatively few peptide vaccine studies to date fully implement end-to-end generative artificial intelligence frameworks coupled to iterative experimental validation. Many published case studies instead represent transitional pipelines, in which classical immunoinformatics and predictive modeling are combined with emerging generative concepts or structure-aware design principles. These examples remain highly informative, as they illustrate both the practical constraints currently limiting fully generative vaccine design and the incremental steps by which generative approaches are being integrated into real-world vaccine development workflows.

The practical impact of generative AI, systems biology, and iterative experimental validation is best illustrated through concrete applications. Although the field is still emerging, several recent studies and translational pipelines demonstrate how integrated frameworks can accelerate the design and refinement of peptide-based vaccines. These examples illustrate how integrated computational and experimental strategies are moving from proof-of-concept toward real-world vaccine development. They also highlight recurrent challenges, including limited experimental datasets, variability across models, and the need to explicitly measure functional immune outcomes.

### 6.1. Infectious Disease Vaccines: Multi-Epitope Design and Translational Validation

In infectious disease research, peptide-based vaccines have been explored both as stand-alone immunogens and as components of multi-epitope constructs designed to broaden coverage and enhance immune robustness. Integrated workflows commonly begin with in silico selection or generation of epitopes, followed by multi-objective filtering for HLA binding, antigen processing, toxicity, and conservation. However, the translational strength of these approaches depends on downstream experimental validation, particularly assays demonstrating functional cellular responses and, ideally, protection in vivo.

A representative example of an end-to-end pipeline integrating computational design with experimental testing is the immunoinformatic-guided development of multi-epitope peptide vaccine candidates that progressed to in vivo immunogenicity studies. In canine circovirus models, peptide constructs designed computationally were experimentally validated in vivo, supporting the feasibility of using immunoinformatic-guided peptide engineering to elicit measurable immune responses [91]. Similar integrated approaches have been applied to dengue virus, where cross-reactive, multi-serotype peptide candidates were designed and subsequently validated in vivo, reinforcing the importance of experimental confirmation beyond predicted binding [92].

These examples illustrate a key theme: in infectious disease contexts, computational design substantially accelerates candidate generation, but vaccine relevance is established only when peptides demonstrate functional immune activation and protection in vivo.

### 6.2. Therapeutic Peptide Vaccines: Cancer Immunotherapy as a Driver of Innovation

Therapeutic vaccines, particularly in oncology, have become a major driver of peptide vaccine innovation. Here, the goal is not sterilizing protection, but the induction of potent cytotoxic and helper T-cell responses capable of targeting tumor cells. This setting is well suited to AI-driven design because tumor neoantigens are patient-specific and require rapid generation and prioritization.

Recent work has demonstrated the use of in silico pipelines to design multi-epitope peptide vaccines targeting cancer-associated antigens, followed by experimental verification of immunological activity. For example, a computationally designed multi-epitope peptide vaccine targeting triple-negative breast cancer incorporated predicted epitopes, linker optimization, and immunogenicity assessment, with subsequent in vitro validation demonstrating immune activation in human-derived experimental systems [93]. These developments align naturally with precision vaccinology frameworks, where immunogen design is explicitly conditioned on host HLA profiles and immune-response objectives [84]. Early first-in-human personalized neoantigen vaccination studies in melanoma have provided direct clinical proof of concept for this paradigm, demonstrating the feasibility of inducing robust, poly-specific CD4^+^ and CD8^+^ T-cell responses through computationally prioritized peptides followed by experimental validation. In particular, independent clinical trials showed that personalized peptide and RNA-based neoantigen vaccines elicited durable tumor-specific immune responses and evidence of clinical benefit in patients with high-risk melanoma [85,86].

Cancer vaccine research also emphasizes the broader methodological point that vaccine success cannot be inferred from binding predictions alone. The most meaningful endpoints are functional: cytokine profiles, cytotoxic activity, tumor control, and durable memory responses.

### 6.3. Tick and Vector-Borne Vaccinology: Opportunities for Peptide Vaccines and Systems-Guided Design

Vector-borne diseases represent a particularly compelling context for systems-guided and AI-assisted vaccine development. Ticks transmit a broad range of pathogens, including intracellular bacteria and viruses, and tick-host–pathogen interactions involve complex molecular cross-talk. This complexity makes the tick vaccine field a natural application domain for systems biology approaches and integrative vaccine design.

Anti-tick vaccine development has already produced promising antigen families, including Subolesin, and has advanced toward real-world deployment in veterinary settings. Notably, field evaluation of Subolesin-based anti-tick vaccine demonstrated effectiveness and safety under operational conditions, offering rare and highly valuable evidence of translational feasibility in this domain [94]. Broader strategic analyses have highlighted both the scientific potential and the implementation challenges of anti-tick vaccination programs, particularly in regions where cattle ticks impose major economic and health burdens [95].

From a systems perspective, tick-borne disease biology further underscores why purely sequence-based predictions are insufficient. Host immune responses to tick feeding and pathogen transmission involve coordinated regulatory networks spanning innate immunity, inflammation, tissue remodeling, and adaptive immune activation. Systems biology frameworks explicitly map these interactions and help identify intervention points that may not be obvious from antigen sequence alone [96]. This is directly relevant to peptide vaccine design: by integrating proteomic and transcriptomic signatures of tick-host–pathogen interaction, computational pipelines can prioritize epitopes derived from conserved tick proteins or from pathogen factors critical for transmission.

**Figure 5:**
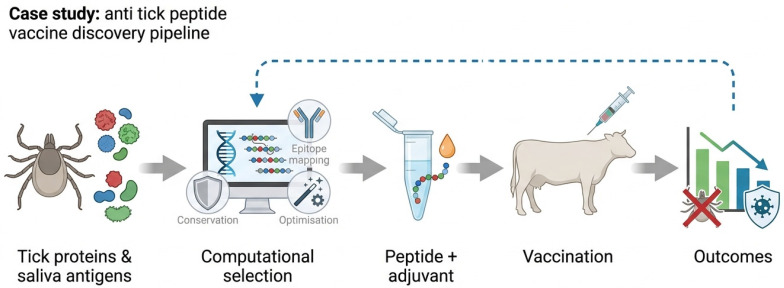
Systems-guided pipeline for anti-tick peptide vaccine development. Schematic overview of an integrated workflow combining multi-omics characterization of tick-host–pathogen interactions, computational antigen prioritization, peptide design and experimental validation. The pipeline illustrates how systems biology and iterative testing are used to identify conserved tick antigens, optimize peptide immunogens and evaluate functional outcomes such as reduced tick infestation, feeding success and pathogen transmission.

Importantly, tick vaccine development also illustrates the need to explicitly consider vaccine formulation and delivery. Anti-tick immune protection may require strong adjuvantation, sustained antigen exposure, and specific immune polarization to achieve meaningful reductions in tick infestation and pathogen transmission. These requirements emphasize why experimental validation must extend beyond immunogenicity to measurable functional outcomes in vivo, including reduced tick attachment, feeding success, fecundity, or pathogen load. Figure 5 illustrates an example of an integrated systems-guided pipeline for anti-tick peptide vaccine development.

### 6.4. Lessons Across Case Studies: Common Bottlenecks and Translational Criteria

Across infectious disease, cancer, and vector-borne vaccinology, several shared principles emerge:

First, computational pipelines are most valuable when they are explicitly embedded in a vaccine-development framework, rather than treated as stand-alone prediction exercises. Second, the most convincing translational evidence comes from studies that integrate multiple experimental layers, including in vitro functional assays and in vivo immunogenicity or protection. Third, systems-level data increasingly provide the mechanistic bridge between peptide properties and whole-organism vaccine outcomes, enabling the design of immunogens that align more closely with protective immune signatures [60,90].

Together, these case studies show that the convergence of generative AI, systems biology, and experimental immunology is beginning to deliver practical, validated vaccine candidates. The next challenge is to scale these approaches, standardize experimental benchmarks, and build richer training datasets that directly encode functional immune outcomes (Table 5).

Together, these case studies highlight both the promise and the current limitations of generative approaches in peptide vaccine development, underscoring the need for richer experimental datasets and tighter integration between sequence generation and functional immune validation.

## 7. Challenges, Limitations and Future Perspectives

Although the convergence of generative AI, systems biology, and experimental immunology offers a compelling framework for next-generation peptide vaccine design, several limitations currently constrain translation into robust prophylactic and therapeutic vaccines. These challenges span data, modeling, experimental validation, and implementation, and are particularly important to address for the field to progress beyond proof-of-concept studies.

### 7.1. Data Scarcity, Bias, and the Gap Between Binding and Protection

Generative models require large, well-curated datasets to learn meaningful sequence–function relationships. However, for peptide vaccines, experimentally annotated datasets that include functional immune outcomes remain limited. Many public resources are enriched for binding measurements (e.g., MHC affinity) rather than protection, durability, or safety, even though binding is only a necessary but insufficient step toward vaccine efficacy [15,62]. As a result, AI models trained on binding-dominant datasets may optimize for proxies that correlate imperfectly with real immune protection.

A particularly severe limitation concerns the availability of experimentally validated data linking peptide-MHC complexes to T cell receptor recognition and functional immunogenicity. While large datasets exist for peptide-MHC binding, far fewer studies provide paired information on TCR engagement, T cell activation, and downstream functional outcomes. As a result, models trained exclusively on binding affinity are poorly constrained with respect to the biological determinants of T cell immunogenicity. Recent analyses have emphasized that immunogenicity emerges from a multi-step process involving antigen processing, MHC presentation, TCR recognition, and cellular context, and that failure to capture this full cascade leads to systematic overestimation of candidate quality. The scarcity, fragmentation, and experimental heterogeneity of MHC-TCR immunogenicity datasets therefore represent a major bottleneck for both predictive and generative modeling, limiting the reliability of de novo peptide vaccine design [97].

In addition, training and evaluation practices can artificially inflate reported performance when homologous proteins are split across training, validation and test sets, leading to homology-driven data leakage and overestimation of generalization. To mitigate this, datasets should be partitioned using homology-aware clustering so that closely related sequences do not appear in different splits. A second persistent limitation is the definition of negative examples: absence of evidence for immunogenicity does not necessarily imply a true negative, which motivates strategies such as positive-unlabeled learning and the systematic generation of experimentally validated negatives through controlled immunization or functional assays [98,99,100].

Bias is also a major concern. Immunological datasets over-represent certain pathogens, alleles, and experimental systems, producing skewed model behavior and potentially misleading “high-confidence” predictions outside well-sampled domains. This issue is particularly relevant for neglected tropical diseases and vector-borne pathogens, including tick-borne systems, where experimental datasets are comparatively sparse and biological complexity is high [89].

### 7.2. Multi-Objective Optimization Remains Difficult in Real Vaccine Constraints

Peptide vaccine candidates must satisfy multiple constraints simultaneously: immunogenic potency, population coverage, manufacturability, solubility, stability, low toxicity, appropriate immune polarization, and compatibility with adjuvants and delivery systems. Multi-objective optimization can be approached through reinforcement learning or constrained generative sampling, but constructing biologically meaningful reward functions remains difficult. Poorly designed objectives can lead to “reward hacking” in which models generate sequences that score well computationally but fail experimentally.

Structural constraints pose additional complexity. Peptides are often conformationally flexible, and structural presentation can determine whether an epitope is accessible to antibodies or efficiently presented by MHC. Structure-aware generation methods, including diffusion models and protein design algorithms, offer a promising direction [80,81] but they remain computationally intensive and require careful experimental grounding.

### 7.3. Experimental Validation Remains the Central Bottleneck

An additional practical constraint concerns the time scales associated with iterative design–test–learn workflows that include in vivo experimentation. While in silico generation and in vitro screening can often be completed on the order of days to weeks, in vivo immunogenicity and protection studies typically require substantially longer timelines, ranging from several weeks to months per iteration. As a consequence, fully closed-loop pipelines incorporating repeated in vivo feedback are unlikely to support large numbers of iterations. In practice, vaccine development workflows are more likely to rely on a small number of strategically placed in vivo experiments, informed by extensive in vitro filtering and systems-level analysis, rather than on rapid multi-cycle in vivo optimization.

Even when computational candidates appear highly promising, the transition from prediction to vaccine requires robust experimental validation. In vitro assays provide valuable early screening, but they cannot fully capture organismal complexity, including antigen processing, adjuvant-driven innate immune activation, tissue-specific effects, or long-term memory formation. Consequently, in vivo studies remain essential for determining protective efficacy and safety.

This requirement directly aligns with systems vaccinology frameworks, which emphasize integrated measurement of immune responses and identification of correlates of protection [60,90]. However, in vivo experimentation is costly, slow, and ethically constrained, and the lack of standardized benchmarks across laboratories limits reproducibility and comparability.

From a translational perspective, one of the most valuable future contributions to the field would be the creation of shared benchmark datasets linking peptide sequences to standardized in vitro and in vivo immune outcomes. Such datasets would enable robust training and evaluation of generative AI models under realistic vaccine development conditions.

### 7.4. Closing the Loop: Active Learning and Integrated Experimental Platforms

A key future direction is the development of closed-loop pipelines that integrate computational generation, high-throughput in vitro testing, and model updating via active learning. In this paradigm, experimental results are continuously incorporated to refine model parameters and improve candidate quality over successive iterations. This approach can substantially reduce the number of experiments required to identify effective vaccine peptides and can increase model reliability in underexplored antigen domains.

Systems-level immune profiling can further strengthen these feedback loops by providing high-dimensional readouts—such as transcriptomic signatures, cytokine modules, and immune-cell phenotypes—that connect peptide features to immune mechanisms and durability. These strategies represent a practical route toward data-efficient, biologically grounded generative vaccinology [84].

### 7.5. Translation and Implementation: Formulation, Delivery, and Real-World Constraints

For peptide vaccines, translation also depends heavily on formulation and delivery, including adjuvant selection, carrier conjugation, nanoparticle presentation, and dosing strategy. These factors profoundly influence immune polarization and durability, and they must be integrated into the design loop rather than treated as downstream “add-ons.” This is particularly relevant for veterinary and vector-borne vaccine applications, where field conditions, host variability, and logistical constraints can strongly influence effectiveness.

Anti-tick vaccination illustrates both the promise and complexity of real-world deployment. Field evaluation of Subolesin-based vaccines demonstrates translational feasibility but also underscores that efficacy depends on formulation, host management practices, and ecological context [94,95]. Future generative AI pipelines should therefore consider not only immunogenicity but also formulation compatibility and field-deployable performance.

### 7.6. Future Perspective: Toward Precision Peptide Vaccinology

Looking forward, the integration of generative AI, systems biology, and experimental validation points toward precision peptide vaccinology, where vaccine candidates can be rapidly designed for specific populations, host genotypes, or disease contexts. This is particularly relevant for cancer vaccines and for pathogens with rapid antigenic evolution, where speed and adaptability are critical.

In the longer term, the field may move toward semi-autonomous vaccine discovery systems combining generative models, automated screening, and systems-level immune readouts. Achieving this vision will require rigorous benchmarking, transparent model interpretability, and a sustained focus on experimental validation as the ultimate measure of vaccine relevance (Table 6).

## 8. Conclusions

Peptide-based vaccines are evolving from epitope prediction plus assembly toward an engineering discipline grounded in systems immunology, generative design and iterative experimentation. What is transforming the field is not only the ability to generate candidates, but the capacity to connect design objectives to experimentally measurable correlates and to update models based on biological outcomes. The most effective programs will therefore treat computation and immunology as a single integrated loop, using standardized in vitro functional gates and carefully chosen in vivo endpoints to progressively reduce attrition.

In the near term, the most realistic gains will come from constraint-first design, where processing, presentation, HLA coverage, structural context and formulation compatibility are built into design objectives rather than imposed after generation. This is particularly important for multi-epitope constructs, where epitope ordering, linker composition and competitive effects can determine whether a design remains functional. Progress will also depend on the availability of richer, more comparable experimental datasets with quantitative functional endpoints and complete metadata, including systematic reporting of negative results. When clinical translation is the aim, human relevant systems, diverse HLA panels, ex vivo PBMC assays and appropriate humanized platforms will be essential to reduce the gap between laboratory readouts and real-world performance.

Generative AI will not replace immunology, but it can make peptide vaccine development substantially more efficient by prioritizing experiments, exploring design space more intelligently and learning from failures. Under this iterative and evidence-grounded trajectory, peptide-based platforms are well positioned to become more routinely successful across both prophylactic and therapeutic applications, including complex contexts such as vector-borne disease control.

## Figures and Tables

**Table 1:** Experimental advances driving the transition from reverse vaccinology to systems-guided antigen selection. Summary of key experimental platforms that have expanded the antigen discovery paradigm from sequence-based filtering toward systems-level, network-informed antigen selection. The table highlights the types of immune information generated by each platform, their contributions to vaccine candidate prioritization, and representative original studies that illustrate how experimental data increasingly shape rational vaccine design.

Experimental Platform	Type of Immune Information Obtained	Contribution to Antigen Selection	Representative Original Studies
Immunopeptidomics (LC–MS/MS)	Naturally processed and presented MHC ligands	Direct measurement of true antigenic landscape; validation of epitope processing and presentation	[41,54]
Single cell transcriptomics	Cell state resolution and immune activation programs	Identification of immune response signatures and correlates of protection	[55]
Immune repertoire sequencing	Clonal expansion and antigen-driven selection	Tracking of adaptive immune dynamics and vaccine induced clonotypes	[56,57]
Systems vaccinology	Integrated immune signatures following vaccination	Prediction of vaccine efficacy, durability and reactogenicity	[58,59]
Multi-omics immune profiling	Coordinated regulation across molecular layers	Contextualization of antigen selection within immune networks	[60,61]

**Table 2:** Conceptual and experimental levels of vaccine evaluation. Comparison of antigenicity, immunogenicity and protective efficacy, highlighting the corresponding biological questions, dominant computational proxies and experimental validation strategies required at each level. The table illustrates why predictive performance at early stages does not necessarily translate into protective immunity and underscores the need for integrated, multi-scale validation pipelines in peptide vaccine development.

Level	Core Biological Question	Dominant Computational Proxies	Required Experimental Validation
Antigenicity	Can the peptide bind an immune receptor (TCR or BCR)?	MHC binding affinity, epitope prediction, structural docking	MHC binding assays, structural validation
Immunogenicity	Can the peptide trigger and sustain an immune response?	Epitope density, predicted processing, population HLA coverage	Antigen processing assays, T-cell activation, cytokine profiling
Protective efficacy	Does the induced immune response prevent disease or infection?	Composite scores, surrogate immune correlates	Animal challenge models, protection studies, durability and safety assessments

**Table 3:** Generative AI architectures and control objectives in peptide vaccine design. Overview of the main generative model classes and their contributions to controlled immunogen engineering.

Model Class	Design Capability	Conditioning Objectives	Key Advantages for Vaccines
VAE	Continuous latent design	Immunogenicity, stability	Well suited for smooth optimization and diversity
Transformer	Sequence generation	HLA binding, length, composition	Effective modeling of long-range dependencies
Reinforcement learning	Multi-objective optimization	MHC affinity, toxicity, solubility	Supports explicit control of design trade-offs
Diffusion models	Structure-aware generation	Geometry, epitope display	Enables high structural fidelity under appropriate constraints
Hybrid pipelines	End-to-end design	Immunogenic + manufacturability	Facilitates translational readiness when integrated end-to-end

**Table 4:** Multi-scale experimental validation in peptide vaccine design. Integration of computational design and experimental evaluation across molecular, cellular and organismal levels within the in silico–in vitro–in vivo continuum.

Stage	Primary Objectives	Key Methods	Design Decisions Informed
In silico	Candidate generation and prioritization	Generative AI, epitope prediction, population modeling	Sequence selection, construct optimization
In vitro	Functional immunological screening	MHC binding, antigen processing, T-cell activation, cytokine assays	Candidate filtering, model retraining
In vivo	Protective efficacy and safety	Animal challenge, immune memory analysis, toxicity studies	Final candidate selection, formulation refinement
Systems feedback	Model refinement and learning	Multi-omics integration, immune signature analysis	Feature weighting, objective redefinition

**Table 5:** Representative applications of integrated peptide vaccine design. Examples of how computational modeling, systems biology and experimental validation converge to generate translational peptide vaccine candidates across infectious disease, oncology and vector-borne vaccinology.

Application Domain	Target	Computational Strategy	Experimental Validation	Key Outcome
Infectious disease	Canine circovirus	Multi-epitope immunoinformatics	In vivo immunogenicity	Measurable immune responses
Infectious disease	Dengue virus	Cross-serotype epitope design	In vivo protection	Broad immune activation
Cancer immunotherapy	Triple-negative breast cancer	Multi-epitope peptide design	In vitro functional assays	Tumor-directed immune activation
Vector-borne disease	Tick Subolesin	Systems-guided antigen selection	Field vaccination studies	Reduced tick infestation

**Table 6:** Key challenges and future directions in generative peptide vaccinology. Summary of major technical and translational limitations currently facing peptide vaccine development and the corresponding strategies proposed to overcome them within integrated generative and systems-driven frameworks.

Domain	Current Challenge	Emerging Solution	Translational Impact
Data	Scarcity of datasets linking sequences to protection	Shared benchmark datasets with functional immune endpoints	Improved model training and evaluation
Modeling	Bias and overfitting to binding proxies	Multi-objective generative optimization	More reliable vaccine candidates
Validation	Limited standardization across laboratories	Harmonized in vitro and in vivo benchmarks	Higher reproducibility
Design	Weak integration of formulation constraints	Constraint-first generative pipelines	Better real-world performance
Deployment	Limited adaptability to evolving pathogens	Active learning and closed loop pipelines	Rapid response vaccination

## Data Availability

No new data were created.
